# Triglyceride-glucose index is associated with lupus nephritis and gender disparity in systemic lupus erythematosus patients

**DOI:** 10.3389/fmed.2025.1579187

**Published:** 2025-05-30

**Authors:** Shuhan Lin, Liangying Tao, Fan Yang, Ruilu Shu, Weimeng Cheng, Lihui Wen, Huayong Zhang

**Affiliations:** ^1^Department of Rheumatology and Immunology, Nanjing Drum Tower Hospital, Affiliated Hospital of Medical School, Nanjing University, Nanjing, China; ^2^Department of Emergency Medicine, Yangzhou Traditional Chinese Medical Hospital, Yangzhou, China; ^3^Department of Health Management Center, Nanjing Drum Tower Hospital, The Affiliated Hospital of Nanjing University Medical School, Nanjing, China; ^4^Department of Cardiology, Nanjing Drum Tower Hospital, The Affiliated Hospital of Nanjing University Medical School, Nanjing, China

**Keywords:** lupus nephritis, triglyceride-glucose index, triglyceride, systemic lupus erythematosus, gender

## Abstract

**Objective:**

Recent studies investigated that triglyceride glucose (TyG) index and triglyceride (TG) are associated with an elevated likelihood of developing and worsening chronic kidney disease (CKD). We aimed to evaluate the correlation between the TyG index and TG with lupus nephritis (LN), respectively, and explore its value in monitoring LN.

**Methods:**

1,192 Systemic Lupus Erythematosus (SLE) patients were involved in this cross-sectional investigation. The presence or absence of LN was used to divide the individuals involved into two distinct categories. Multivariate logistic regression, restricted cubic spline, and subgroup analyses were applied to explore the connection between the TyG index and TG with LN, respectively.

**Results:**

According to the study, the TyG index and TG were dose-dependently positively correlated with LN. After accounting for additional factors, each standard deviation of an upsurge in the TyG index and TG corresponded to a higher risk for LN by 36.4 and 34%, respectively. Besides, the adjusted ORs (with 95% CIs) for LN were precisely 1.625 (1.097, 2.405) and 1.756 (1.193, 2.585) when comparing the highest tertile to the lowest tertile of the TyG index and TG, respectively. Additionally, both TyG index and TG were significantly positively correlated with LN in age and body mass index (BMI) subgroups, and these two indicators were independently associated with LN in female SLE patients but not in male SLE patients, respectively.

**Conclusion:**

Both the elevated TyG index and TG were linked to LN on their own and gender disparity in SLE patients, which suggests that the TyG index and TG could be beneficial in the early screening for those with LN.

## Highlights

- We found that both TyG index and TG levels were linearly correlated with LN.
- Gender difference in the association between TyG index and TG with LN.
- As low-cost, routinely available indicators, the TyG index and TG facilitate screening for LN in hospitalized patients with SLE.

## 1 Introduction

Systemic lupus erythematosus (SLE) is an autoimmune disease with an incidence of 30–50 per 100,000 individuals in China (1, 2). SLE has a varied clinical course and a broad spectrum of organ presentations, frequently causing kidney damage (3). One of the most severe organ symptoms of the autoimmune condition SLE is lupus nephritis (LN), a form of glomerulonephritis (3, 4). Compared to patients without LN, patients with renal involvement have more excellent rates of morbidity and mortality (5). Early diagnosis and immunosuppressive therapy are critical for the prognosis of LN.

Insulin resistance (IR) is an early metabolic change in patients suffering from chronic kidney disease (CKD) and may accelerate kidney failure (6, 7). The logarithmically derived product of fasting triglyceride and glucose levels, known as the triglyceride-glucose (TyG) index, has been proposed as a straightforward indicator of IR (8). Recent clinical research has discovered a link between the TyG index and kidney disease in various individuals (9, 10). However, the connection between the TyG index and LN remains unexplored. Whether the TyG index contributes to the development of LN warrants investigation.

Patients with SLE are often accompanied by lipid metabolism disorders. Recently, a series of studies have reported a specific relationship between triglyceride levels, disease activity scores, and inflammatory factors in patients with SLE, providing a basis for diagnosing and evaluating SLE (11, 12). Nevertheless, research concerning triglyceride levels among lupus nephritis patients is scarce, with limited sample sizes, necessitating further investigation.

Therefore, we aimed to explore the correlation between the TyG index and TG with LN in individuals with SLE to offer insights into the early detection, monitoring, and management of LN.

## 2 Methods

### 2.1 Participants

The present investigation was conducted at a single medical center in East China, encompassing 2,109 SLE patients who were initially admitted between July 2014 and February 2023. The diagnosis of SLE was made by the standards for systemic lupus erythematosus established by the *Ad Hoc* Committee of the American College of Rheumatology (13). Exclusion criteria included: (a) pre-existing comorbidities: hypertension, diabetes mellitus, metabolic syndrome or coronary artery disease; (b) cardiovascular risk factors: active smoking, body mass index (BMI) > 25 kg/m^2^, history of hyperlipidemia, or current use of lipid-lowering medications; (c) coexisting autoimmune diseases other than Sjögren’s syndrome; (d) incomplete clinical or laboratory data. A total of 1,192 SLE patients were ultimately enrolled, comprising 627 cases without lupus nephritis (Non-LN group) and 565 cases with biopsy-proven or clinically confirmed lupus nephritis (LN group). The control group consisted of randomly selected subjects who visited the Nanjing Drum Tower Hospital for a health check-up and had no evidence of illness during the same period.

### 2.2 Data collection and associated definitions

Patient demographic information, laboratory indicators and medication data were obtained via a database built by an electronic medical record system called YiduCloud (Beijing) Technology Ltd. Demographic information included age, sex, body mass index (BMI), systolic blood pressure (SBP) and diastolic blood pressure (DBP). Laboratory indicators included triglyceride (TG), high-density lipoprotein (HDL), low-density lipoprotein (LDL), total cholesterol (TC), apolipoprotein A1 (ApoA1), apolipoprotein B (ApoB), fasting blood glucose (FBG), white blood cell count (WBC), hemoglobin (Hb), platelet (PLT), albumin (ALB), 24-h urine protein, urine protein/creatinine ratio, creatinine (Cr), uric acid (UA), blood BUN nitrogen (BUN), creatinine (Cr), estimated glomerular filtration rate (eGFR), urinary leukocyte, urinary erythrocyte, complement component 3 (C3), complement component 4 (C4), anti-dsDNA antibodies, anti-nucleosome antibodies (AnuA), anti-histone antibodies (AHA). Medication data included the use of glucocorticoids [including current use (yes/no) and daily prednisone equivalent dose (mg/ day)], cyclophosphamide, hydroxychloroquine and mycophenolate mofetil. LN is clinically characterized by renal involvement in SLE, manifesting as proteinuria, hematuria, or cellular casts, or confirmed by pathological evidence. According to the diagnostic criteria established by the American College of Rheumatology (ACR), LN is defined by fulfilling at least one of the following: (1) persistent proteinuria above 0.5 g/d or more extraordinary than 3 + by dipstick or urine protein/creatinine ratio above 0.5 g/g (2) active urinary sediment (>5 RBCs/high-power field [hpf], >5 white blood cells [WBCs]/hpf in the absence of infection, or cellular casts limited to RBC or WBC casts) (3) a renal biopsy sample demonstrating immune complex–mediated glomerulonephritis compatible with LN (14). In this study, the diagnosis of LN cases was based on a combination of clinical criteria (ACR guidelines) and renal biopsy findings. For non-biopsied cases, diagnosis was established through laboratory indicators such as persistent proteinuria (>0.5 g/24 h) or a urine protein-to-creatinine ratio >0.5 g/g. The histopathological classification of LN was based on the 2003 International Society of Nephrology/Renal Pathology Society (ISN/RPS) criteria. This system categorizes LN into six principal classes: Class I (minimal mesangial), Class II (mesangial proliferative), Class III (focal), Class IV (diffuse), Class V (membranous), and Class VI (advanced sclerotic), with additional evaluation of activity index (AI) and chronicity index (CI) to assess acute and chronic renal damage. However, due to the retrospective nature of this study and incomplete archival records of renal biopsies, ISN/RPS subclassification was not systematically applied to all LN cases (15). The TyG index was calculated as ln [TG (mg/dL) × FBG (mg/dL)/2] (16). GC dose stratification was performed: <7.5 mg/day (low-dose), 7.5–30 mg/day (medium-dose), >30 mg/day (high-dose). Methylprednisolone pulse therapy was defined as intravenous methylprednisolone ≥200 mg/d for 1–3 days.

### 2.3 Statistical analyses

Continuous parameters were presented as mean ± standard deviation (SD) or median (25th–75th percentile), while categorical variables are generally represented using frequency (%). The chi-squared test, or Fisher’s exact test, assessed the disparities among categorical variables. In contrast, the Student *t*-test, or Mann–Whitney test, was utilized for continuous variables. TyG index and TG were divided into three quantiles, with the lowest quantile (T1) as the reference group. Logistic regression analysis assessed the association between the TyG index, TG, and LN, utilizing the covariate-adjusted odds ratios (ORs) and 95% confidence intervals (CIs). Due to the presence of multicollinearity between independent variables, we finally retained only LDL for the relevant indicators of lipid profiles. Finally, adjusted covariates included age, SBP, DBP, LDL, Hb, Alb, UA, BUN, Cr, C3, C4, eGFR, urinary leukocyte, urinary erythrocyte, anti-dsDNA and use of glucocorticoids, cyclophosphamide and mycophenolate mofetil drugs. The restricted cubic spline approach was employed to investigate the dose–response relationship further. To determine if the correlations between the TyG index, TG, and LN altered depending on the status of the potential variables (age, sex, eGFR, anti-dsDNA, BMI and use of GCs), we conducted subgroup and interaction analyses. R version 4.2.2 and SPSS version 27 were used to analyze the data. A *p* value<0.05 was considered to indicate statistical significance.

## 3 Results

### 3.1 Baseline characteristics

Participants were categorized into two groups based on the presence or absence of LN (Table 1). The mean age was 33.7 ± 7.63 y in the controls group, 39.04 ± 15.10 y in the Non-LN group, and 36.54 ± 14.02 y in the LN group. LN occurred at an earlier age than non-lupus nephritis (*p* < 0.001) in 1192 SLE patients investigated. Of the 588 healthy subjects, 502 (85.4%) were women, while there were 555 (88.5%) women in the Non-LN group, and 498 (88.1%) women in the LN group. There was no difference among patients when we controlled for gender (*p* = 0.84). No significant difference was observed in the three groups in terms of age, FBG, AnuA, AHA, and use of hydroxychloroquine (*p* > 0.05). In contrast to patients without LN, those with LN preferred to have a higher SBP, DBP, TG, TyG index, LDL, ApoA1, ApoB, TC, UA, BUN, Cr, Urinary leukocyte, Urinary erythrocyte level; a higher rate of Anti-dsDNA positivity and use of high dose GC, cyclophosphamide and mycophenolate mofetil; and significantly lower levels of C3, C4, Hb, Alb, and eGFR (all *p* < 0.05).

**Table 1:** Comparison of clinical data and laboratory measurements among the groups.

Variables	Control group (*n* = 588)	Non-LN group (*n* = 627)	LN group (*n* = 565)	*p* value (Non-LN vs. LN)
Female (%)	502 (85.4%)	555 (88.5%)	498 (88.1%)	0.84
Age, years	33.7 ± 7.63	39.04 ± 15.10^△^	36.54 ± 14.02^△^	0.005^**^
BMI, kg/m2	21.64 [19.86, 23.09]	20.51 [18.9, 22.48]^△^	20.83 [19.09, 22.87]^△^	0.063
SBP, mmHg	116 [108, 125]	114 [105, 125]^△^	120 [111, 130]^△^	< 0.001^***^
DBP, mmHg	70 [64, 77]	72 [65, 80]^△^	76 [68.5, 84]^△^	< 0.001^***^
Laboratory results				
TG, mmol/L	1.125 [0.81, 1.45]	1.25 [0.88, 1.7]^△^	1.69 [1.22, 2.42]^△^	< 0.001^***^
TyG index	8.27 ± 0.42	8.41 ± 0.52^△^	8.73 ± 0.54^△^	< 0.001^***^
HDL, mmol/L	1.18 [0.94, 1.46]	1.09 [0.85, 1.37]^△^	1.11 [0.83, 1.44]^△^	0.627
LDL, mmol/L	2.27 [1.85, 2.63]	2.11 [1.69, 2.57]^△^	2.53 [1.82, 3.45]^△^	< 0.001^***^
ApoA1, g/L	1.02 [0.88, 1.2]	0.92 [0.77, 1.1]^△^	0.96 [0.77, 1.19]^△^	0.041^*^
ApoB, g/L	0.69 [0.59, 0.81]	0.69 [0.56, 0.83]	0.85 [0.66, 1.08]^△^	< 0.001^***^
TC, mmol/L	4.08 [3.56, 4.55]	3.86 [3.32, 4.51]^△^	4.6 [3.62, 5.65]^△^	< 0.001^***^
FBG, mmol/L	4.5 [4.14, 4.93]	4.41 [4, 5.02]	4.4 [4.01, 5.07]	0.838
UA, umol/L	274 [230, 331]	278 [221, 348]	365 [274.5, 470]^△^	< 0.001^***^
BUN, mmol/L	4.6 [3.7, 5.575]	4.7 [3.7, 6.2]^△^	6.7 [4.7, 10.15]^△^	< 0.001^***^
Cr, umol/L	52 [45, 61]	48 [41, 58]^△^	60 [46, 96]^△^	< 0.001^***^
C3, g/L	1.07 [0.95, 1.21]	0.77 [0.55, 0.99]^△^	0.64 [0.42, 0.89]^△^	< 0.001^***^
C4, g/L	0.23 [0.18, 0.3]	0.13 [0.06, 0.2]^△^	0.1 [0.05, 0.18]^△^	0.005^**^
Hb, g/L	124 [115, 132]	111 [96, 124]^△^	98 [80.5, 114]^△^	< 0.001^***^
WBC, 10^9/L	5.1 [4.1, 6.3]	4.5 [3.2, 6.3]^△^	4.8 [3.2, 7.1]	0.092
Plt, 10^9/L	193.5 [158.25, 236.75]	168 [110, 228]^△^	166 [100.5, 229]^△^	0.514
Alb, g/L	39 [37.3, 40.7]	36.5 [33.4, 39.1]^△^	31.5 [27, 35.2]^△^	< 0.001^***^
eGFR, ml/min	119.05 [100.58, 137.6]	140.7 [111.4, 168.2]^△^	109.8 [63.75, 151.4]^△^	< 0.001^***^
Urinary leukocyte, cells/μL	6.8 [2, 22]	8 [3, 25.3]^△^	16 [7, 56.5]^△^	< 0.001^***^
Urinary erythrocyte, cells/μL	3 [1.3, 8]	4 [1.3, 11.9]^△^	15 [4, 70.5]^△^	< 0.001^***^
Anti-dsDNA Ab positive (%)	-	240 (38.3%)	253 (44.8%)	0.023^*^
AnuA positive (%)	-	185 (29.5%)	178 (31.5%)	0.454
AHA positive (%)	-	189 (30.1%)	174 (30.8%)	0.807
Medication use (%)				
Glucocorticoids				< 0.001^***^
Low dose GC	-	103 (16.4%)	52 (9.2%)	a
Medium dose GC	-	286 (45.6%)	154 (27.3%)	a
High dose GC	-	187 (29.8%)	335 (59.3%)	b^*^
Methylprednisolone pulse therapy	-	20 (3.2%)	12 (2.1%)	a
CTX	-	98 (15.6%)	160 (28.3%)	< 0.001^***^
MMF	-	117 (18.7%)	200 (35.4%)	< 0.001^***^
HCQ	-	493 (78.6%)	434 (76.8%)	0.452

Values were expressed as mean ± SD, median (25th–75th percentile), or frequency (percent) as appropriate. BMI, Body Mass Index; SBP, systolic blood pressure; DBP, diastolic blood pressure; TG, triglyceride; TyG index, Triglyceride glucose index; HDL, high-density lipoprotein; LDL, low-density lipoprotein; ApoA1, apolipoprotein A1; ApoB, apolipoprotein B; TC, Total cholesterol; FBG, Fasting blood glucose; UA, Uric acid; Cr, creatinine; C3, complement C3; C4, complement C4; Hb, hemoglobin; WBC, White blood cell count; Plt, platelet; Alb, albumin; eGFR, estimated glomerular filtration rate; Anti-dsDNA Ab, anti-double-stranded deoxyribonucleic acid antibody; AnuA, anti-nucleosome antibodies; AHA, anti-histone antibodies; GC, Glucocorticoid; CTX, cyclophosphamide; MMF, mycophenolate mofetil; HCQ, hydroxychloroquine.* *p* < 0.05, **p < 0.01, ***p < 0.001, Δ Compared with control group, *p* < 0.05.

### 3.2 Association of the TyG index and TG with LN

The relationships between two variables with LN were examined using multiple logistic regression analyses. The findings of these studies are presented in Table 2. The TyG index exhibited a statistically significant and positive correlation with LN in the unadjusted model, as was TG. Following multivariable adjustment, it was observed that each SD increase in the TyG index (SD = 0.55) was associated with a 36.4% higher risk of LN (*p* < 0.001), while a 1-SD increase in TG (SD = 1.07) corresponded to a 34% elevated risk of LN (*p* = 0.001). When comparing the highest to lowest tertiles of the TyG and TG, the adjusted ORs (95% CI) for LN were 1.625 (1.097, 2.405) and 1.756 (1.193, 2.585), respectively. These results indicate a statistically significant trend (*p* < 0.05).

**Table 2:** Odds ratios (95% CIs) of LN founded on continuous or tertiles of indicators.

Variables	Crude model OR (95% CI)	*p* value	Model A OR (95% CI)	*p* value
TyG (Per 1 SD increase)	1.917 (1.681, 2.185)	< 0.001^***^	1.364 (1.155, 1.611)	< 0.001^***^
Tertiles of TyG				
T1 (< 8.34)	1.00 (reference)		1.00 (reference)	
T2 (8.34–8.78)	2.007 (1.504, 2.678)	< 0.001^***^	1.275 (0.884, 1.84)	0.193
T3 (> 8.78)	3.821 (2.841, 5.138)	< 0.001^***^	1.625 (1.097, 2.405)	0.015^*^
*p* for trend		< 0.001^***^		0.015^*^
TG (Per 1 SD increase)	2.033 (1.735, 2.382)	< 0.001^***^	1.34 (1.12, 1.604)	0.001^**^
Tertiles of TG				
T1 (< 1.17)	1.00 (reference)		1.00 (reference)	
T2 (1.17–1.77)	2.089 (1.561, 2.795)	< 0.001^***^	1.333 (0.924, 1.923)	0.124
T3 (> 1.77)	4.278 (3.175, 5.764)	< 0.001^***^	1.756 (1.193, 2.585)	0.004^**^
*p* for trend		< 0.001^***^		0.004^**^

Model A was adjusted for age, SBP, DBP, LDL, Hb, Alb, UA, BUN, Cr, C3, C4, eGFR, urinary leukocyte, urinary erythrocyte, anti-dsDNA and use of glucocorticoids, cyclophosphamide and mycophenolate mofetil drugs.

**Figure 1:**
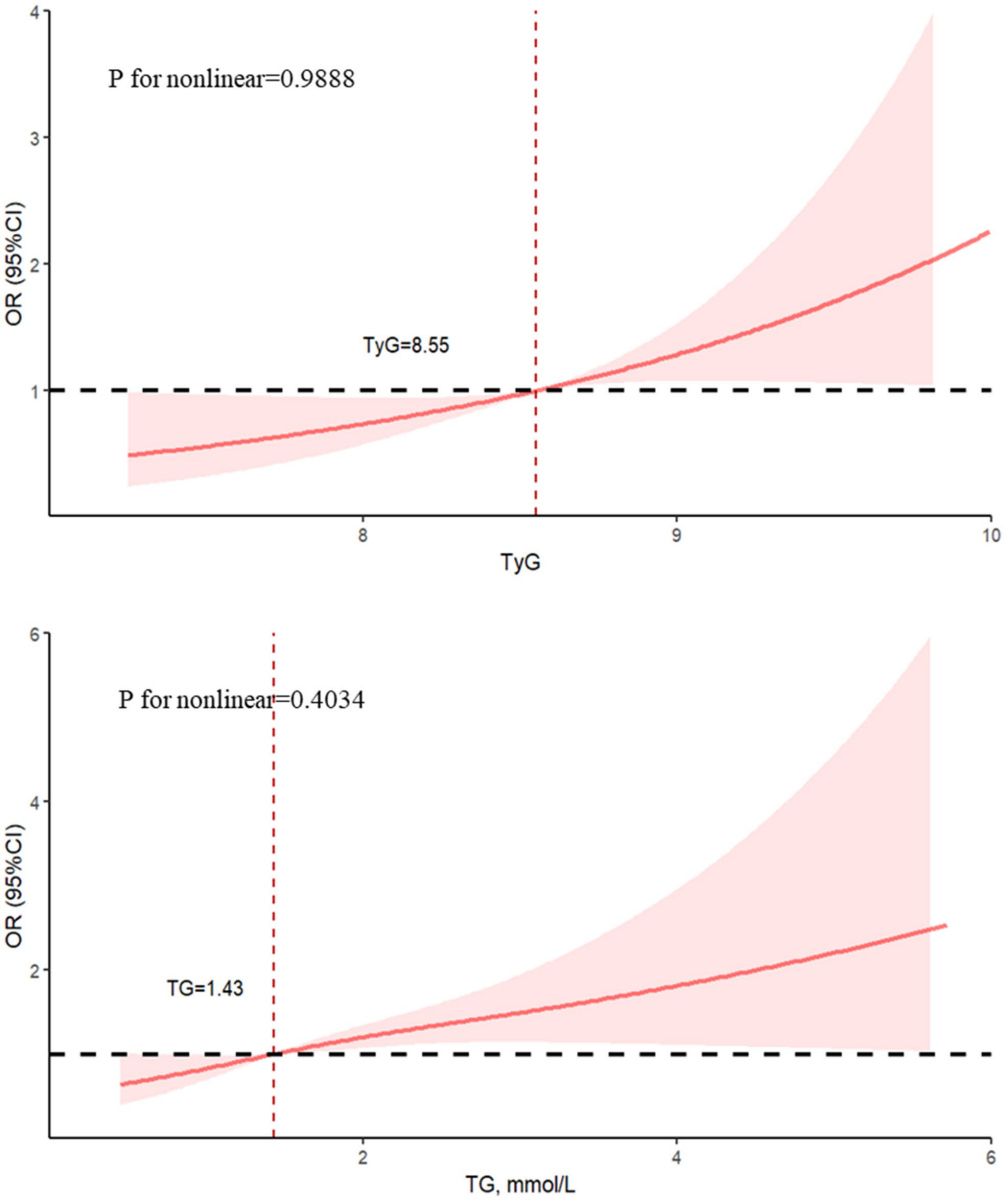
Restricted cubic spline regression was used to calculate the odds ratios shown as solid lines and 95% confidence intervals shown as shaded areas. Age, SBP, DBP, LDL, Hb, Alb, UA, BUN, Cr, C3, C4, eGFR, urinary leukocyte, urinary erythrocyte, anti-dsDNA and use of glucocorticoids, cyclophosphamide and mycophenolate mofetil drugs were among the adjustment factors.

Additional analyses utilizing restricted cubic splines further confirmed the positively linear relationship between the TyG index and TG with LN, respectively (*p* for nonlinear > 0.05), as depicted in Figure 1.

### 3.3 Subgroup analyses

**Figure 2:**
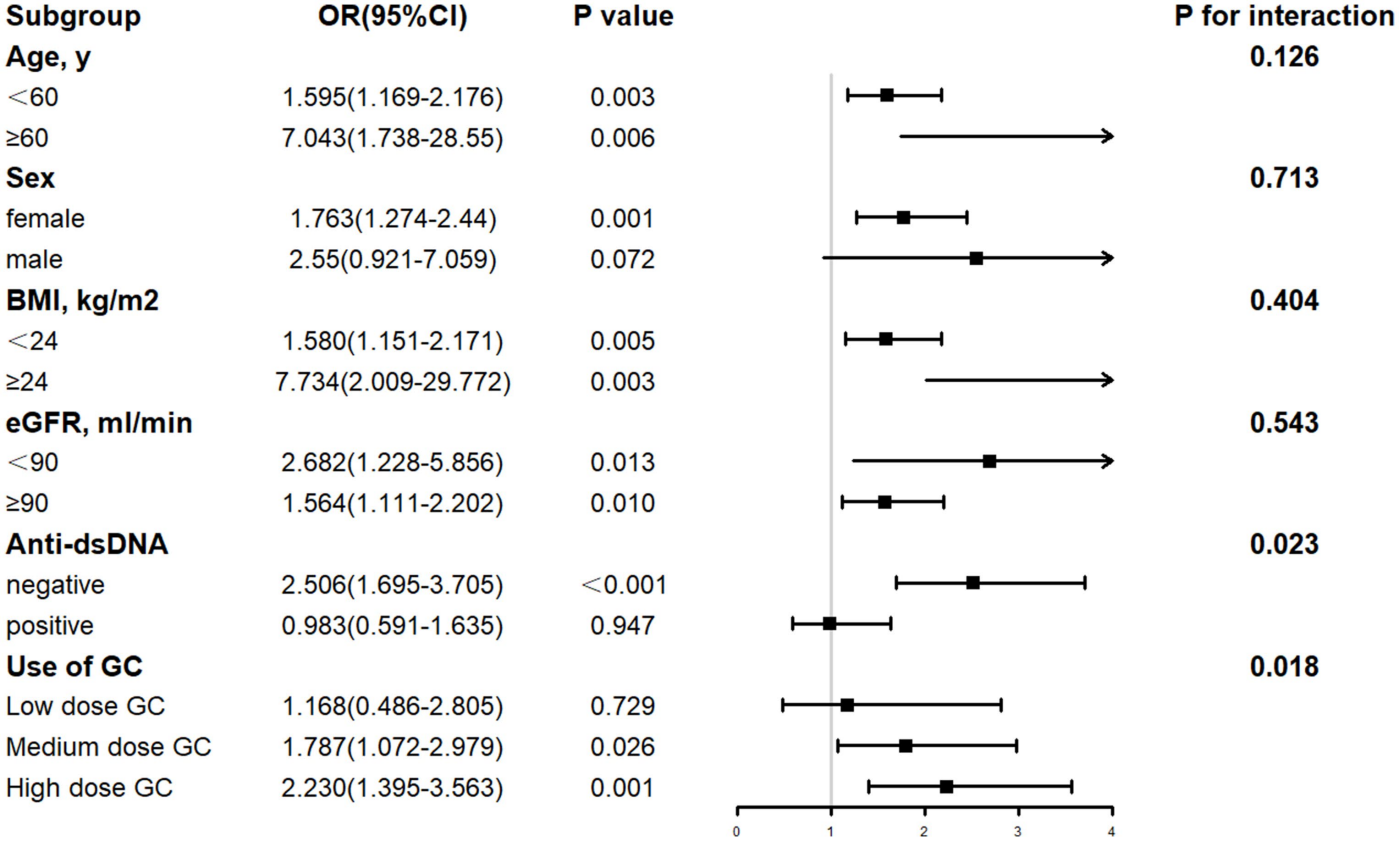
Subgroup analysis for the link between the TyG and LN. Age, SBP, DBP, LDL, Hb, Alb, UA, BUN, Cr, C3, C4, eGFR, urinary leukocyte, urinary erythrocyte, anti-dsDNA and use of glucocorticoids, cyclophosphamide and mycophenolate mofetil drugs were among the adjustment factors.

**Figure 3:**
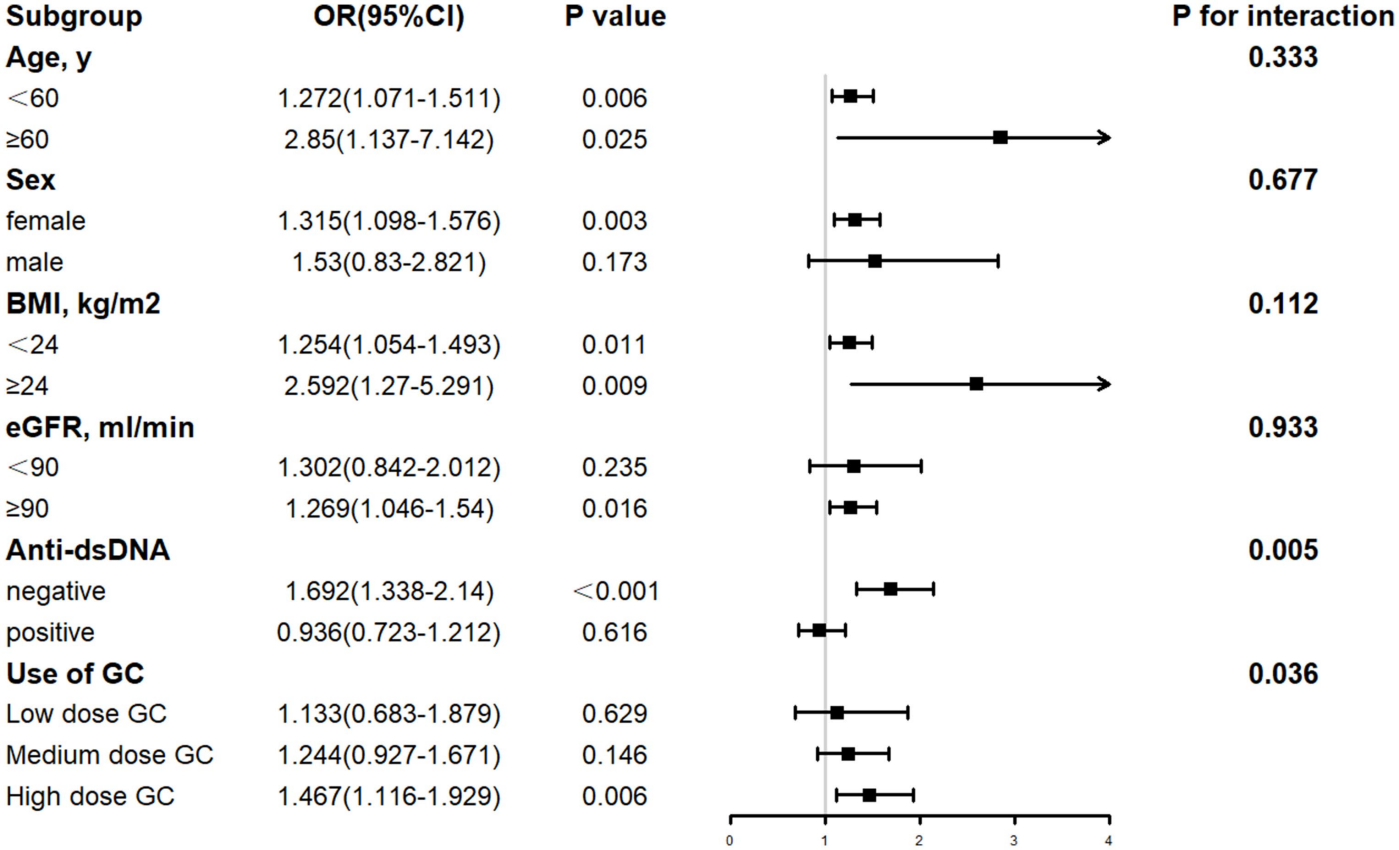
Subgroup analysis for the link between the TG and LN. Age, SBP, DBP, LDL, Hb, Alb, UA, BUN, Cr, C3, C4, eGFR, urinary leukocyte, urinary erythrocyte, anti-dsDNA and use of glucocorticoids, cyclophosphamide and mycophenolate mofetil drugs were among the adjustment factrs.

The forest diagram (Figures 2, 3) showed that after controlling for covariates, the TyG index was significantly correlated with LN in subgroups stratified by age, eGFR, and BMI (*p* < 0.05). In contrast, TG level was significantly correlated with LN in subgroups stratified by age and BMI (*p* < 0.05). In the associations of the TyG index and TG with LN, no significant interactions were observed for age, sex, eGFR, or BMI (*p* for interaction > 0.05). Significant heterogeneity was identified for anti-dsDNA status and GC dose (*p* for interaction < 0.05), suggesting differential effects of TyG and TG on LN risk in these subgroups. Elevated TyG index and TG levels demonstrated significant associations with LN in female SLE patients, anti-dsDNA-negative individuals, and those receiving high-dose glucocorticoid therapy (all *p* < 0.05). In contrast, no significant correlations were detected in male SLE patients, those with anti-dsDNA positivity, or individuals treated with low-to-medium-dose glucocorticoids (*p* < 0.05). Notably, elevated TG levels were significantly associated with LN in SLE patients with preserved renal function (eGFR >90 mL/min; *p* < 0.05), whereas these associations lacked statistical significance in patients with reduced renal function (eGFR <90 mL/min; *p* < 0.05).

## 4 Discussion

The present cross-sectional investigation shows that LN in Chinese patients with SLE is linked to the TyG index and TG. The limited cubic spline curve shows these two indexes have a linear relationship with LN. In addition, we also observed gender differences in the association between TyG index and TG with LN through subgroup analysis.

The TyG index and LN have not been studied before, so this is an important starting step. Because it is a straightforward and effective technique of IR (17), the TyG index has been widely utilized in epidemiology investigations. IR has been linked to reduced kidney function (18), which may cause or speed up the progression of CKD through microvascular damage, chronic inflammation, and oxidative stress (19–21). A cross-sectional investigation illustrated a connection between the TyG index and early kidney impairment in individuals with hypertension (22). Furthermore, two recent studies revealed that a positive association between TyG index and diabetic nephropathy in hospitalized subjects with type 2 diabetes (23, 24).

This study presents the initial evidence of a linear correlation between the TyG index and LN. Nevertheless, the underlying mechanisms responsible for this association have yet to be fully elucidated. The pathogenesis of IR in individuals with CKD entails a complex interaction among various factors, including inflammation, dysregulation of adipokines, lipotoxicity, and uremic toxins (18, 25). In LN, IR may be associated with renal tubular interstitial fibrosis and arteriolar sclerosis (26). Meanwhile, hyperinsulinemia stimulates sodium reabsorption and induces glomerular hyperfiltration, leading to kidney damage (27). Furthermore, our research revealed that those with a high TyG index demonstrated elevated uric acid concentrations, blood lipids, and blood pressure. Multiple studies have proposed a notable correlation between the TyG index and hyperlipidemia, hypertension, and hyperuricemia (28, 29). These comorbidities have the potential to exacerbate renal injury in individuals with SLE.

LN is a significant determinant impacting the prognosis of individuals diagnosed with systemic lupus erythematosus. For patients with LN, persistent dyslipidemia is a significant factor in developing chronic renal impairment (30, 31). Hence, it is imperative to prioritize the management of dyslipidemia in clinical practice and adopt a proactive approach toward its treatment. Our finding aligns with the results reported in prior research (31–33), indicating an apparent positive correlation between TG levels and the advancement of LN. We also found a positive dose–response relationship between TG levels and lupus nephritis, which enriched previous studies. Dyslipoproteinemia, characterized by hypertriglyceridemia, is prevalent among lupus patients (34). Hypertriglyceridemia can result in harm to renal endothelial cells, hence inducing renal injury through the process of hyperfiltration of the glomerular filtration barrier (35). Simultaneously, during the initial phase of kidney injury, IR may contribute to the overproduction of very low-density lipoprotein cholesterol, resulting in hypertriglyceridemia (36), exacerbating the progression of kidney damage.

Subgroup analysis revealed that in SLE patients with preserved renal function (eGFR ≥90 mL/min), elevated TG levels demonstrated a significant association with LN risk (*p* < 0.05). However, this association lost statistical significance in patients with impaired renal function (eGFR <90 mL/min). Furthermore, the TyG index exhibited heterogeneous associations with LN across eGFR subgroups. The decline in eGFR is often accompanied by a complex interplay of uremic toxin accumulation, chronic inflammatory states, and dysregulated lipid metabolism (37, 38). At this stage, coexisting pathological factors, such as hypertension and anemia, may obscure the independent effects of TG and TyG index on LN, leading to attenuated associations. Therefore, in patients with impaired renal function, comprehensive management of uremia-related complications is warranted, rather than relying solely on metabolic indicators to guide therapeutic strategies.

We found that increasing TyG index and TG levels were still significantly and independently linked with LN in age and BMI subgroups. In addition, female patients’ TyG index and TG levels were related to LN but not in male patients. In mouse models of lupus, female mice are more likely to develop glomerulonephritis (39). Studies have found that the decrease of estrogen in postmenopausal women leads to a decrease in insulin sensitivity and is prone to insulin resistance (40, 41), which also affects lipid metabolism (42). In our study, the lupus nephritis group had an average age of 36.5 years, which cannot account for the population in this age group, so further research is needed.

The differential associations of TyG index and TG levels with LN risk across anti-dsDNA status and GC dose subgroups suggest distinct pathophysiological pathways. In anti-dsDNA-negative patients, significant associations between TyG/TG and LN may reflect a predominant role of metabolic dysregulation in driving renal injury, as opposed to immune complex-mediated mechanisms, such as complement activation, inflammatory cascades, that dominate in anti-dsDNA-positive individuals (43). It implies that metabolic biomarkers like TyG and TG hold greater predictive value in LN patients lacking classical autoimmune drivers. Furthermore, the dose-dependent strengthening of TyG/TG-LN associations in high-dose GC users (TyG OR = 2.230, *p* = 0.001; TG OR = 1.467, *p* = 0.006) aligns with the known metabolic perturbations induced by high-dose GC therapy, including exacerbated insulin resistance and dyslipidemia (44). Conversely, the lack of significance in low-to-medium-dose GC subgroups may stem from milder metabolic disruption or confounding by concurrent immunosuppressive therapies that attenuate the independent effects of metabolic factors.

This study has several limitations that should be acknowledged. First, while the cross-sectional design facilitated efficient association analysis, it inherently precludes causal interpretations. We cannot establish whether TyG/TG elevation predates LN development or arises secondary to renal impairment, nor can we characterize dynamic TyG/TG changes across disease stages (pre-LN, active phase, remission). This study also did not systematically document dynamic changes in anti-dsDNA antibody levels before and after GC therapy, which may affect the subgroup analysis results. High-dose GC therapy might suppress antibody production, potentially biasing the observed association between anti-dsDNA status and LN. Second, diagnostic reliance on clinical-laboratory criteria without routine renal biopsy verification risks underdiagnosing histopathologically active but clinically silent LN, while preventing exploration of TyG/TG variations among pathological subtypes with distinct metabolic features. And because electronic health record data are collected retrospectively, there are inconsistencies in the archiving of urinary sediment reports. Third, single-center recruitment, despite its large sample size, limits generalizability due to geographical/genetic homogeneity and potential selection bias. Finally, our findings leave critical mechanistic and clinical gaps unanswered: the biological pathways connecting TyG/TG to LN pathogenesis remain unelucidated. To overcome these limitations, prospective studies should longitudinally track TyG/TG trajectories pre- and post-LN diagnosis and temporal changes in anti-dsDNA antibody levels and metabolic indicators (TyG/TG) to elucidate the regulatory effects of GC therapy on their interplay. Additionally, integrating renal biopsy histopathological classification (WHO/ISN-RPS criteria) could further explore interactions between metabolic and immune pathways across distinct LN subtypes. Mechanistic investigations can delineate how TyG/TG participates in LN-related metabolic-inflammatory interplay, and ethnically diverse validation cohorts with rigorous confounder adjustments are needed to establish universal applicability.

## 5 Conclusion

In conclusion, this study indicates a favorable correlation involving the TyG index and LN, demonstrating an independent dose-dependent relationship, as well as TG. In addition, our finding demonstrated a gender difference in the association between the TyG index and TG with LN. The TyG index and TG are easy-to-detect biochemical indicators in clinical practice, which can have potential value in the early screening of patients with LN.

## Data Availability

The original contributions presented in the study are included in the article/Supplementary material, further inquiries can be directed to the corresponding authors.
